# Promotion of Healthy Nutrition and Physical Activity Lifestyles for Teenagers: A Systematic Literature Review of The Current Methodologies

**DOI:** 10.3390/jpm10010012

**Published:** 2020-03-01

**Authors:** María Vanessa Villasana, Ivan Miguel Pires, Juliana Sá, Nuno M. Garcia, Eftim Zdravevski, Ivan Chorbev, Petre Lameski, Francisco Flórez-Revuelta

**Affiliations:** 1 Faculty of Health Sciences, Universidade da Beira Interior, 6200-506 Covilhã, Portugal; julianasa@fcsaude.ubi.pt; 2 Instituto de Telecomunicações, Universidade da Beira Interior, 6200-001 Covilhã, Portugal; impires@it.ubi.pt (I.M.P.); ngarcia@di.ubi.pt (N.M.G.); 3 Polytechnic Institute of Viseu, 3504-510 Viseu, Portugal; 4 Hospital Center of Cova da Beira, 6200-251 Covilhã, Portugal; 5 Faculty of Computer Science and Engineering, University Ss Cyril and Methodius, 1000 Skopje, North Macedonia; eftim.zdravevski@finki.ukim.mk (E.Z.); ivan.chorbev@finki.ukim.mk (I.C.); petre.lameski@finki.ukim.mk (P.L.); 6 Department of Computer Technology, Universidad de Alicante, P.O. Box 99, E-03080 Alicante, Spain; francisco.florez@ua.es

**Keywords:** diet, nutrition, physical activity, education, methods, teenagers, healthy lifestyles, gamification, health, mobile devices

## Abstract

Amid obesity problems in the young population and apparent trends of spending a significant amount of time in a stationary position, promoting healthy nutrition and physical activities to teenagers is becoming increasingly important. It can rely on different methodologies, including a paper diary and mobile applications. However, the widespread use of mobile applications by teenagers suggests that they could be a more suitable tool for this purpose. This paper reviews the methodologies for promoting physical activities to healthy teenagers explored in different studies, excluding the analysis of different diseases. We found only nine studies working with teenagers and mobile applications to promote active lifestyles, including the focus on nutrition and physical activity. Studies report using different techniques to captivate the teenagers, including questionnaires and gamification techniques. We identified the common features used in different studies, which are: paper diary, diet diary, exercise diary, notifications, diet plan, physical activity registration, gamification, smoking cessation, pictures, game, and SMS, among others.

## 1. Introduction

Due to the emergence and improvement of several technological devices [1,2,3,4], e.g., smartphones, smartwatches, and tablets, one of the most relevant effects consist of the appearance of sedentary lifestyles [5,6]. Consequently, several health problems are appearing, including obesity and cardiovascular diseases, among others [7,8,9,10,11]. However, these devices can also promote healthy lifestyles by encouraging correct levels of physical activity and nutrition [12,13,14,15].

Previous works related to nutrition and physical activity [16,17] are associated with the development of solutions for promoting healthy lifestyles of teenagers. Earlier works focused on the measurement of energy consumption [18] and calorie expenditure during physical activities [19], which helps in promoting healthy lifestyles. Such approaches utilize sensors and equipment related to the implementation of Ambient Assisted Living (AAL) systems and could be similarly used to create personal digital life coaches [20].

Recently, the monitoring of daily activities using mobile devices has been widely researched for different healthcare or leisure related topics, for instance, jogging periods estimation in adolescents based on wearables [21]. Research has shown that personalized approaches and systematic feature engineering and selection can help in improving the activity recognition accuracy [22,23,24]. In addition to using inertial sensors (i.e., accelerometers and gyroscopes) embedded in mobile devices, acoustic sensors can augment and improve the activity and environment recognition [25,26]. However, these methods have several limitations that should be considered during the development of these systems, such as availability of the sensors, weather conditions, battery lifetime, limited power processing, and memory capabilities, among others [27,28].

Even though all previously mentioned studies report high activity recognition accuracy on mobile devices, teenagers find it hard to accept and adopt these methods. Gamification, the use of game elements in non-game settings, is a recent approach to increase the popularity of learning. There are studies on this topic that were mainly focused on educational settings. A recent review article [29] performed an in-depth literature review on adaptive gamification in education to provide a synthesis of current trends and developments in this field. It identified that gamification is more and more used in education to increase learner motivation, engagement, and performance. In particular, the game elements should be tailored to learners, and adaptive gamification should be used.

Similarly, [30] illustrated the application of gamification in the educational context by providing examples from the literature. In summary, successful gamification approaches include design elements including points, levels/stages, badges, leaderboards, prizes, progress bars, storyline, and feedback. Another review study of gamification in education is [31]. According to this review, the main driver for the adoption of gamification techniques is the enhancement of motivation and engagement in learning tasks, i.e., to make learning more attractive, captivating and, ultimately, compelling. Gamification techniques are being adopted to support learning in a variety of educational contexts and subject areas, but also to address behaviors such as collaboration, creativity, and self-guided study. It identified that most works employing gamification are at the university level and in professional learning environments. 

A most similar study on gamification and promoting healthy lifestyles in teenagers was CoviHealth [32]. It defined a taxonomy combining gamification elements and considering nutrition, physical activity, and questionnaires to improve the lifestyles in teenagers.

This paper goes beyond this and reviews studies related to the promotion of healthy nutrition and physical activity habits in teenagers using mobile devices. It researches the use of mobile applications associated with a healthy diet and physical activity habits by teenagers aged between 13 and 18 years old. In this research, we found studies published between 2009 and 2018 and against our expectations, gamification is not one of the most used features. The authors mainly used questionnaires to evaluate the knowledge of teenagers.

The remainder of the paper is structured as follows. Section 2 presents the methodology, including research questions, inclusion and exclusion criteria, search strategy, and extraction of study characteristics. The results are shown in Section 3, which are discussed in Section 4. Finally, the conclusions are presented in Section 5.

## 2. Methodology

### 2.1. Research Questions

The main questions of this review were as follows: (RQ1) Which are the most commonly used methodologies relying on mobile applications to educate young people for healthy lifestyle habits? (RQ2) How do mobile applications promote healthy nutrition and physical activity habits for teenagers? (RQ3) Do young people respond with positive feedback to mobile applications for nutrition and physical activity?

### 2.2. Inclusion Criteria

Articles about studies related to the nutrition and physical activity habits were included in this review based on the following criteria: (1) studies related to teenagers; (2) studies related to people aged between 13 and 18 years old; (3) studies related to fitness metrics, gamification, goals, nutrition, or physical activity purposes; (4) studies published between 2009 and 2019; and (5) studies written in English.

### 2.3. Exclusion Criteria

Articles were excluded based on the following exclusion criteria: (1) studies related to medical pathologies; (2) studies not associated with health subject; (3) studies only focused in one genre; (4) studies that are literature reviews; (5) studies related to sex education; (6) studies related to specific subjects of medicine, such as psychiatry, rheumatology, and dermatology; (7) studies related to drugs; and (8) studies related to oral hygiene.

### 2.4. Search Strategy

Based on the research questions and inclusion and exclusion criteria, exhaustive research was performed in the following databases: Springer, IEEE Xplore, and PubMed. These databases include study in different subjects, including medicine, computer science, sociology, psychology, and others. We used an Natural Language Processing (NLP)-based framework to automatically search the digital libraries and identify potentially relevant papers [33]. The framework also automatically removed duplicates, as well as papers according to our inclusion and exclusion criteria. The remaining studies were analyzed by eight researchers (M.V., I.P., J.S., N.G., E.Z., I.C., P.L., and F.R.), who all agreed with the analysis. The studies were examined to mainly identify the different strategies used in studies primarily related to nutrition, physical activity, health, and gamification, and its suitability for the application with teenagers.

### 2.5. Extraction of Study Characteristics

For the extraction of the characteristics, several data were extracted from the analyzed papers and presented in Table 1 and Table 2. These are the year of publication, the population for the study, the purpose of the study, devices used, use of questionnaires, the inclusion of gamification, methods used, incorporation of nutrition subject, integration of physical activity subject, and study outcomes of the study. We additionally emailed the corresponding author of each paper to obtain more details related to the development of a new methodology to implement with young people.

## 3. Results

**Figure 1:**
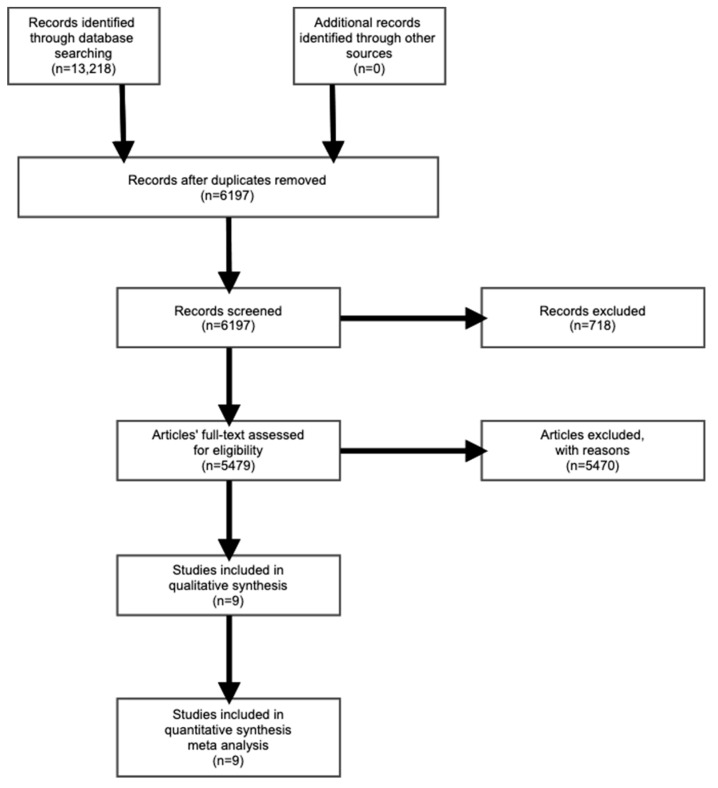
PRISMA flow diagram of identification and inclusion of papers.

As presented in Figure 1, this review identified 13,218 scientific articles; 7012 were removed, because they were duplicated between the different databases. The remaining 6197 papers were evaluated based on title, abstract, and keywords, where 718 papers were excluded. After that, the full text of the remaining 5479 articles was assessed, resulting in the exclusion of 5470 papers that matched the exclusion criteria or did not match the inclusion criteria. Finally, the remaining nine papers were included in the quantitative and qualitative synthesis. Thus, our systematic review analyzed nine articles.

This review only synthesizes the different research works, where the interested readers should read the original research works to obtain more detailed information about the methodologies analyzed in this review. Table 1 shows the year of publication, population, the purpose of the study, devices, methods, and the inclusion of gamification, nutrition, and physical activity methods. Table 2 shows the outcomes of different research studies. All studies analyzed in Table 1 included the use of questionnaires as an assessment method. Following the studies analyzed, only one study was published in 2009 (11.12%), two studies were in 2013 (22.22%), two studies were in 2014 (22.22%), two studies were in 2017 (22.22%) and, finally, two studies were in 2018 (22.22%). All papers are based on the use of mobile applications for different assessments. Still, one article (11.12%) combined the use of a mobile application with other primate methods, *i.e.,* the use of paper. Following the inclusion of gamification methods, 78% of the analyzed studies did not use gamification, and only two articles (22.22%) used gamification as a method of captivating young people. Following the inclusion of a nutrition module, four studies (44.44%) addressed the subject of nutrition and healthy eating. Finally, a physical activity module was also included in four articles (44.44%), promoting physical activity in teenagers. Finally, analyzing the population involved in the different studies, three studies (33.33%) included people more than 18 years old, where one study (11.12%) included people up to 21 years old. The remaining people were aged between 13 and 18 years old, of which four studies (44.44%) involved people that were 13 years old, six studies (66.67%) included teenagers that were 14, 15, and 18 years old, seven studies (77.78%) included people that were 17 years old, and, finally, eight studies (88.89%) included people that were 16 years old. The different studies were from around the globe; two studies were placed in New York (22.22%), four studies in Europe (44.44%), *i.e.*, United Kingdom, Belgium, Finland, and the Netherlands, and the remaining three studies (33.34%) were placed in countries from different continents, including Korea (Asia), Brazil (South America), and Australia.

### Methodologies Implemented in Different Studies

In [16], the authors implemented a mobile application named “FoodWiz2” to help young people choose healthy lifestyles. This study included 34 young people aged between 16 and 19 years old. It was conducted in Norfolk and Suffolk in the United Kingdom [16]. The concept consisted of the comparison of the record of food intake and exercise performed in a paper diary and a mobile application, completing a questionnaire on the experience of use of tools [16]. The mobile application also included personalized messages related to the user’s activity [16]. Following the questionnaires, it was concluded that a mobile application was preferable for daily logging to a paper diary, improving eating habits with the use of the mobile application [16].

The authors of [34] evaluated the feasibility and impact of a mobile phone intervention that incorporated explicit reflection strategies and implicit rewards during adolescent snack intake. The authors included 988 young people in this study, where 416 received responses and 572 served as controls [34]. Data were collected with the use of questionnaires and mobile application data. The difference in the proportion of healthy snacks, awareness, intention, attitude, self-efficacy, habits, and knowledge were assessed [34]. They had a low adherence and a high dropout throughout the study, having no results that demonstrate high importance in the use of this mobile application, without the intervention of third parties, namely teachers [34].

In [35], the authors evaluated the viability of a mobile application named “Diet-A” to find out the possibility to monitor young people’s diet intake. The study included 35 young people aged between 16 and 18 years [35]. Before and after the intervention, young people were encouraged to record everything they ingested by measuring nutrient intake using 24-h recalls [35]. It was found that young people tend to underreport nutrient intake, with a decrease in sodium and calcium intake between pre- and post-intervention [35]. Analyzing the results, the majority (61, 9%) were satisfied with the use of the mobile application, and 47.7% liked to see personal information about their intake. Additionally, 70% of teenagers thought it was unpleasant to use the mobile application, or they had difficulty in remembering to log the data [35]. In conclusion, this mobile application offers the ability to monitor food intake with real-time feedback [35].

One study [36] aimed to measure its effectiveness in changing smoking behavior in a mirroring approach using a mobile photoaging application named "Smokerface”. This study included young people from 13 to 19 years old, in Itauna, Brazil [36]. At the start, a selfie was taken for each participant [36]. Before 3 and 6 months after the intervention, a tobacco consumption questionnaire was implemented [36]. In a total of 735 students, two groups were made, where one made use of the mobile application, and the other group did not have access to the mobile application [36]. An interactive photograph was taken to a volunteer, projecting it to the classroom with the possibility to change the face according to the smoking and non-smoking time [36]. At the end of the session, students were encouraged to take an anonymous questionnaire based on the five-point Likert scale, regarding motivation to continue non-smoking, to learn about the benefits of non-smoking, and the perceived enjoyment of the intervention [36].

The authors of [37] evaluated access to mobile applications in pediatric clinics by analyzing demographics, smartphone usage, and interest in the use of health-related mobile applications. This study was implemented in the Bronx, New York, where 148 questionnaires were applied in the waiting rooms using the mobile application named “iHealthNYC” [37]. Each quiz had 24 questions [37]. This study showed that caregivers would be more interested in using health-related mobile applications [37].

One study [38] aimed to assess the importance of the flow experience in the evaluation of the game and the design process. It involved 53 teenagers aged between 13 and 15 years old, which played a collaborative game designed to increase collaboration and communication skills [38]. By the end, the experience was based on a questionnaire and the behavior during the game [38].

Another study aimed to understand the health information needed by adolescents during their daily lives and to evaluate how they know the needs [17]. This study included 60 young people from 13–18 years old with a selection based on the Gauss distribution, where 77% of them usually use a smartphone [17]. For this study, the authors gave a smartphone to each participant with unlimited SMS and data for 30 days, where mobile applications related to asthma, obesity, Human Immunodeficiency Virus (HIV), and diet were installed [17]. During the study, messages were sent to the teenagers, asking their health questions each day, the source of information, and whether they had found the answer [17]. This study found that using text messages is an excellent way to get in touch with young people and to understand how they feel about their health [17].

In [39], the authors aimed to evaluate the viability, usability, and ecological validity of a mobile application named “mEMA" to Diet Intake and Physical Activity. They applied this mobile application to 30 Dutch students aged between 16 and 21 years old for seven days. Students used the mobile application with their smartphone. After seven days, an online questionnaire was available. Over the days, a decrease in the compliance rate was verified. The teenagers evaluated the mobile application as viable and usable, demonstrating that the mobile application is useful to assess dietary intake and physical activity behaviors.

The authors of [40] aimed to develop and evaluate a youth mobile phone program to monitor, in real-time, young people’s daily experiences of mood, stress, and coping behaviors. In total, 29 young people entered the project, where 11 analyzed the mobile application, and 18 were monitored for seven days [40]. One mobile application was used to distribute questionnaires with a frequency of four times a day, collecting information on current activity, mood, negative mood response, stress, and use of alcohol and cannabis [40]. Students reported that the mobile application was able to capture their attitude, thoughts, and activities [40].

## 4. Discussion

The use of mobile applications is the primary focus of this review, where the different analyzed studies considered different methodologies to captivate the attention of teenagers. All considered studies used mobile applications and questionnaires to verify the satisfaction of the teenagers in subjects related to Health and Education and to promote the use of the proposed methodologies. To summarize this, Table 3 presents the different features included in each study. Only two of the analyzed studies (22.22%) used a mobile application with questionnaires related to health and education, which were not presented in Table 3 [37,40]. The remaining studies also included other relevant features, where one study (11.12%) used a paper diary combined with a mobile application to compare its use, four studies (44.44%) took into account a diet diary, and only three studies (33.33%) were based in the promotion of the physical activity.

According to our analysis, one study (11.12%) included four features, two studies (22.22%) were composed of five features, one study (11.12%) had six features, two studies (22.22%) included seven features, and nine features were included in one study (11.12%). Only one study (11.12%) proposed a mobile application that provides push notifications to the user. Additionally, four studies (44.44%) offered the possibility to create diet diaries, and three studies (33.33%) allowed the registration of physical activity. The subject "smoking cessation" was presented in only one study (11.12%), where the authors used photography to stimulate the user to stop smoking. In general, the studies used mobile applications, but one of them made use of a game, where the research was related to the analysis and evaluation based on the use of the game. Finally, one study (11.12%) used SMS, in the mobile application, to communicate with the teenagers and to provide reliable information. 

**Figure 2:**
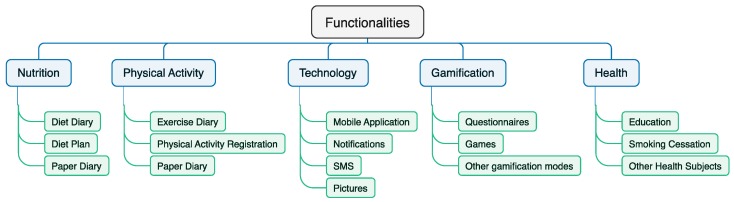
Taxonomy of the features included in the different studies.

After the analysis of the different features present in various studies and based on the finality of each feature, we propose a categorization of them in Figure 2. "Nutrition,” "Physical activity,” "Technology,” "Gamification,” and "Health" are the main categories of the different functionalities analyzed. The feature named as "Paper diary" is included in "Nutrition" and "Physical activity,” because its measurement is performed with basic methods in some studies for the comparison with the use of a mobile application. In conclusion, four studies (44.44%) were composed of features included in "Nutrition,” three studies (33.33%) included features related to "Physical activity,” and all the studies included features related to “Technology,” “Gamification,” and “Health.”

The presented studies have several pros and cons, as shown in Table 4, where the major problem found is the insufficient size of the used datasets because it does not have scientific validity in case it does not have representative data of the entire population. In several instances, the use of mobile technologies and games captivate teenagers to have healthy habits. This study was not focused on different diseases, but it is mainly focused on the promotion of healthy lifestyles. It was proved that the use of gamification with technological devices used daily will captivate teenagers. This study also demonstrates that it is possible to improve the habits of teenagers with mobile devices. 

Regarding the use of gamification, physical activity, and nutrition purposes, only two of the studies analyzed (22.22%) reported the use of gamification. Still, the use of gamification with students is important, and it shows reliable results in the literature [29,30,31].

## 5. Conclusions

This study identified studies with different methodologies for the assessment of the acceptability of the use of mobile applications for the promotion of nutrition and physical activity habits with gamification. Mainly, the various studies used questionnaires for different assessments. However, in general, the number of teenagers recruited for different studies is not enough to obtain results with significance. The reviews that match our search keywords are lower because most of the studies related to health, nutrition, mobile devices, and gamification are mainly related to healthcare problems and not only focused on teenagers’ lifestyles. Additionally, the studies that are not related to health were excluded, as well as studies focused on only one genre. Our research attempted to study nutrition and physical activity habits, excluding the analysis of other healthcare problems, including psychiatry, rheumatology, dermatology, drugs, and oral hygiene. Nine studies were analyzed, where the main findings, answering the previously proposed research questions, are:

RQ1. *Which are the most commonly used methodologies relying on mobile applications to educate young people for healthy lifestyle habits?* Mainly, all studies include the use of a mobile application that was tested over some time, which can be hours or months. Then, the mobile application and its user satisfaction were always evaluated with questionnaires. In some cases, the questionnaire was a part of the use of the mobile application, and it was not explicitly used for the evaluation of the users’ satisfaction.
RQ2. *How do mobile applications promote healthy nutrition and physical activity habits for teenagers?* Nine studies were analyzed, but some of them did not address the subject of nutrition and physical activity. Some of them reported that improvement was verified in diet and nutrition habits. There are some medical benefits in physical activity, including the reduction of obesity. The key to capturing the attention of teenagers was a way of the presentation of the information and content, and not the mobile application itself. This finding is in line with the conclusions of other studies on gamification in education.
RQ3. *Do young people respond with positive feedback to mobile applications for nutrition and physical activity?* All studies considered the use of mobile applications that captured the attention of teenagers. Of the analyzed studies, only six out of nine (66.67%) reported positive feedback by the teenagers. Even though all studies reported high acceptance by the teenagers, the effects of the mobile applications were not verified.

This survey highlighted the use of mobile applications that focused on nutrition and physical activity, where the use of gamification and questionnaires increased the use of these types of mobile applications. The limitations of this study are that many studies are focused on several diseases, and our propose is to analyze teenagers without a focus on different disorders. Other limitations were identified in our review. First, the authors excluded studies that did not include concepts related to nutrition, physical activity, gamification, and mobile devices. Secondly, the authors performed other substantial exclusion, excluding the studies related to medical pathologies, sex education, specific subjects of medicine, drugs, and oral hygiene. The papers were excluded, sequentially, with the analysis of abstract, keywords, and full text of the articles. By the end, only the papers written in English-language were included.

Based on the different analyses, we can conclude that the use of gamification and questionnaires captivates teenagers to use mobile applications for the promotion of healthy and physical nutrition habits. Finally, as future work, the distribution of the mobile application involved in the CoviHealth project in the selected secondary schools for further analysis of the effects of the new functionalities proposed in growing communities. 

## Figures and Tables

**Table 1:** Study analysis.

Paper	Year	Location	Population	Purpose of the Study	Devices	Include Gamification	Methods	Include Nnutrition	Include Physical Activity
Florence et al. [16]	2018	Norfolk and Suffolk, United Kingdom	34 teenagers aged between 16 and 19 years old	It consisted of the use of a mobile application to support healthy lifestyles in teenagers.	Mobile application and paper diary	No	- Record the food intake and exercise in a paper diary; - Use of online questionnaires to describe the experience; - Use of personalized messages in response to the mobile application activity; - Promote healthy nutrition and physical activity habits.	Yes	Yes
De Cock et al. [34]	2018	Flanders, Belgium	988 adolescents aged between 14 and 16 years old	It evaluated the impacts of the use of mobile applications that implements rewarding strategies on food intake.	Mobile application	No	- Use a mobile application for four weeks; - Intend to examine the differences in healthy snacking ratio and key determinants; - It logs the nutrition in the mobile application; - Use of online questionnaires to describe the experience.	Yes	No
Lee et al. [35]	2017	Seoul, Korea	9 male and 24 female high school students aged between 16 and 18 years old	It performed a test with the mobile application named “Diet-A” and examines the monitoring of dietary intake among adolescents.	Mobile application	No	- It was used to record all foods consumed; - The voice and text are the modes used to input the data; - The mobile application measures the nutrients intake; - The consumed values are compared with the standard values in the mobile application used.	Yes	No
Faria et al. [36]	2017	Itauna, Brazil	Regular secondary schools aged between 13 and 19 years old	It used a mobile application named “Smokerface,” where the user takes a selfie and the face changes with the number of years smoking.	Mobile application	Yes	- It measures the effects of the mobile application in smoking habits in secondary schools; - It provides questionnaires about smoking habits; - It has games to encourage to quit smoking; - It includes the photoaging calculation with pictures, including facial changes and others.	No	No
Singh et al. [37]	2014	Bronx, New York	Parents and teenagers aged between 13 and 19 years old	It measured the demographic characteristics of mobile devices, its primary usage, and interest in mobile applications for health.	Mobile application	No	- The participants performed 148 surveys with a 24-item “iHealthNYC” questionnaire; - The use of mobile health applications was demonstrated; - The collected data were statistically analyzed.	No	No
Kiili et al. [38]	2014	Finland	53 teenagers aged between 13 and 15 years old	It implemented a game to teach teamwork, collaborative work, and communication skills to teenagers.	Interactive game with a mobile phone	Yes	- Use of questionnaires; - Use of an interactive game to teach, promoting the cooperation; - Promote physical activity with games.	No	Yes
Rebecca et al. [17]	2013	Bronx, New York	60 adolescents aged between 13 and 18 years	It explained the health information needs of adolescents for their everyday lives.	Mobile application	No	- Use of a mobile application and text messages, asking the following questions: (1) What doubts did you have about your health today? (2) Where did you look for an answer (mobile device, mobile application, online, friend, book, or parent)? (3) Was your question answered, and how? (4) Do you have other questions?	Yes	Yes
Spook et al. [39]	2013	Netherland	44 vocational education students aged between 16 and 21 years old	It examined the use of the mEMA application with Dutch vocational education students.	Mobile application	No	- The test was performed during seven consecutive days; - After the seven days, a questionnaire with multiple-choice questions was available in the mobile application; - It includes physical activity monitoring tools.	No	Yes
Reid et al. [40]	2009	Melbourne, Australia	18 students aged between 14 and 17 years old	It evaluated a mobile application to monitor, in real-time, the levels of stress, mood, and coping behaviors.	Mobile application	No	- It includes questionnaires to captures information on current activity, mood, responses to negative attitude, stresses, and alcohol and cannabis use.	No	No

**Table 2:** Study summaries.

Paper	Outcomes
Florence et al. [16]	The study used a mobile application named "FoodWiz2" to support healthy lifestyles in teenagers. The main problem analyzed in the study is related to the poor quality of the diet of adolescents in the United Kingdom, where adolescents do not consume the recommended amount of fruits, vegetables, and fiber.In contrast, saturated fat and sugar were more than the dietary reference value is lower than the reference value. It measures dietary intake with several approaches, including 24-h recall, food frequency questionnaires, food diaries, or duplicate diet measurement. The mobile application integrates different technologies to present personalized dietary advice to support healthy lifestyles. The analysis of food intake and exercise in teenagers is compared with paper-based approaches to measure the use, acceptability, and perceived effectiveness of a mobile application. The mobile application showed that fruit consumption increased, and the consumption of chocolate snacks and fizzy drinks reduced. Based on the questionnaires, the authors conclude that the use of the mobile application is preferred to the paper diary, improving the diet and exercise levels.
De Cock et al. [34]	This study aimed to evaluate the feasibility and impact of a mobile phone intervention that incorporated explicit reflection strategies and implicit rewards during adolescent snack intake. It was verified that there are no significant positive intervention effects in the healthy snack ration or targeted determinants. The users’ satisfaction with the mobile application with the questionnaires was meagre. Thus, it is possible to conclude that there are only small positive improvements in the snack choices of adolescents.
Lee et al. [35]	It used the mobile application named “Diet-A” with adolescents, executing different actions, including the presentation of questionnaires to the user, the record of all foods consumed, the measurement of the nutrient intake, and the comparison of the values consumed with the standard values. However, the values registered by the users are few, but the study concludes that the values of sodium and calcium decreased during the study. Only 61.9% of the adolescents reported that they were satisfied with the monitoring of food intake, and only 47.7% were happy with the dietary intake. Finally, the mobile application was used to offer the tracking of the dietary intake, but it did not provide reliable information about the food intake of the adolescents.
Faria et al. [36]	People start smoking during adolescence. One of the effects of smoking is getting old faster, as well as other verified health consequences. The authors used a methodology based on a mobile application that was easy to implement, and it had good acceptance by teenagers. The mobile application named “Smokerface” has methods for smoking prevention and smoking cessation. One of the essential techniques of smoking prevention is the photoaging that was used in female adolescents with success. Smoking cessation was also impacted by photoaging, but it also implements games to quit smoking and questionnaires. The mobile application used shows reliable results.
Singh et al. [37]	The study used the mobile application named “iHealthNYC” to provide a questionnaire with 24 questions, measuring the demographic characteristics of the mobile devices. The high use of the mobile application and mobile devices in urban pediatric populations provide patient education, streamlining health care communication and disease management. Based on the analysis of the data acquired, more research is needed to enable the patients to get proper health treatment.
Kiili et al. [38]	It used an interactive game to teach and promote cooperation and physical activity. The most important is to achieve goals with the game. The results showed that the measurement of the flow experience can reveal shortages of the game, and in that way aid the design process. The authors evaluated the quality of the playing experience. Still, the analysis should be extended the study of the game mechanics and audio-visual implementation or complementary research methods.
Rebecca et al. [17]	It used a mobile application and text messages to measure the subjective playing experience. The authors attempted to understand the habits of adolescents in the context of daily activities. The amount of mobile health applications is growing to promote the management of health. To study adolescents, the use of mobile applications and text messages is the most important. In general, the users frequently manage mobile applications, and 90% of the user’s responded to the text messages. The study concluded that the text messages are useful to assess the health behavior of young people. While the authors found some issues in diet and exercise, the technology may help in reducing obesity.
Spook et al. [39]	The mEMA mobile application provided questionnaires with multiple choice questions and physical activity monitoring tools. The test had a duration of 7 days, where only 54% of the users accepted to use the mobile application, and only 71% of the students filled the online questionnaires in the mobile application. The study showed that the mobile application is a usable and ecologically valid tool to measure different behaviors, but compliance is still limited.
Reid et al. [40]	The authors used a mobile application to examine mood, stress, and coping with questionnaires. The mobile application shows surveys, where 94% of the participants reported that it correctly analyzed the attitudes, thoughts, and activities. It has high acceptance because 76% of the questions are correctly filled. Finally, the authors concluded that it captured detailed and exciting qualitative and quantitative data about moods, stresses, coping strategies, and alcohol and cannabis use.

**Table 3:** Distribution of the functionalities of the mobile applications explored in each study.

Studies	Features										
	Paper Diary	Diet Diary	Exercise Diary	Notifications	Diet Plan	Physical Activity Registration	Gamification	Smoking Cessation	Pictures	Game	SMS
Florence et al. [16]	X	X	X	X	X	X					
De Cock et al. [34]		X			X						
Lee et al. [35]		X			X						
Faria et al. [36]							X	X	X		
Kiili et al. [38]			X			X	X			X	
Rebecca et al. [17]											X
Spook et al. [39]		X	X		X	X					
Number of studies	1 (11.1%)	4 (44.4%)	3 (33.3%)	1 (11.1%)	4 (44.4%)	3 (33.3%)	2 (22.2%)	1 (11.1%)	1 (11.1%)	1 (11.1%)	1 (11.1%)

**Table 4:** Analysis of the pros and cons of the different studies.

Study	Pros	Cons
Florence et al. [16]	- It focused on encouraging the improvement of overall diet rather than the control of calories; - It included information from UK food composition tables; - It compared the use of a standard for medical control (paper diary) with new technology; - It increased the awareness of adolescents about the food they eat and the level of activity.	- The study was conducted at a time of high academic pressure, which led participants to stop participating in the survey or inhibited the participants from participating in the study.
Kiili et al. [38]	- It promoted physical activity with games	- Teenagers could not concentrate on learning the game as they spent more time to discover how they controlled their character in the game.
Rebecca et al. [17]	- It used SMS to get young people to raise their response	- The sample was not significantly representative of the different ethnicities; - The accuracy was not reported; - SMS responses may be inaccurate due to various factors, such as stigma; - They found some limitations with technology; - They did not have an artificial intelligence method capable of analyzing and providing feedback about the responses and feedback to the participants.
Faria et al. [36]	- It measured the effectiveness of changing smoking habits; - The mobile application provided educational information; - It made the data interpretable for further intervention.	- The results obtained cannot be generalized to other cultures; - The effects of the cluster cannot be excluded, because the people were always from the same schools; - The methods with contamination limited the intensity of the intervention; - The process for the identification of a smoker may be inadequate.
Spook et al. [39]	- The mobile application provided access to sophisticated health information in real-time; - The data acquired has diversity on social and physical factors of influence; - The mobile application has the potential for further research on complex cognitions and other behaviors.	- The validity of the responses was not validated according to the social desirability; - Some activities discouraged teenagers to answer questionnaires.
Lee et al. [35]	- The study was focused on four pathologies based on nutrition habits, including diabetes, obesity, high blood pressure, and dyslipidemia; - The mobile application helped to improve dietary habits, providing feedback on nutrients, disease risk, and food recommendations; - It allowed teenagers to get input and dietary advice immediately.	- The improvement of the diet with the intervention was not verified; - The sample was tiny, and it is not representative of the population; - The mobile application did not measure physical activity.
Singh et al. [37]	- This study had perspectives of teenagers and adults; - The survey considered the demographic characteristics.	- More research is needed to captivate the patients; - The selected sample may already be skewed; - The questions were not validated.
De Cock et al. [34]	- A gamified mobile application captivates teenagers.	- It was not possible to demonstrate a significant positive impact on the intervention on the healthy snack ratio; - The installation of the mobile application was complicated; - The content and the design of the mobile application should be improved.
Reid et al. [40]	- The questionnaires for the monitoring of moods, stressors, and coping increased the awareness of the problems; - The teenagers adopted a positive manner of problem-solving.	- The sample is not representative of the population studied; - The sample was tiny.
